# Supplementary material for: TRIM27 mediates STAT3 activation at retromer-positive structures to promote colitis and colitis-associated carcinogenesis
Source: Nat Commun. 2018 Aug 24;9:3441. doi: 10.1038/s41467-018-05796-z (PMC6109048; doi:10.1038/s41467-018-05796-z)
Supplement: Supplementary file 1 — Supplementary Information [file 41467_2018_5796_MOESM1_ESM.pdf]

Supplementary Information

TRIM27 mediates STAT3 activation at retromer-positive structures to promote colitis and colitis-associated carcinogenesis

Zhang et al.

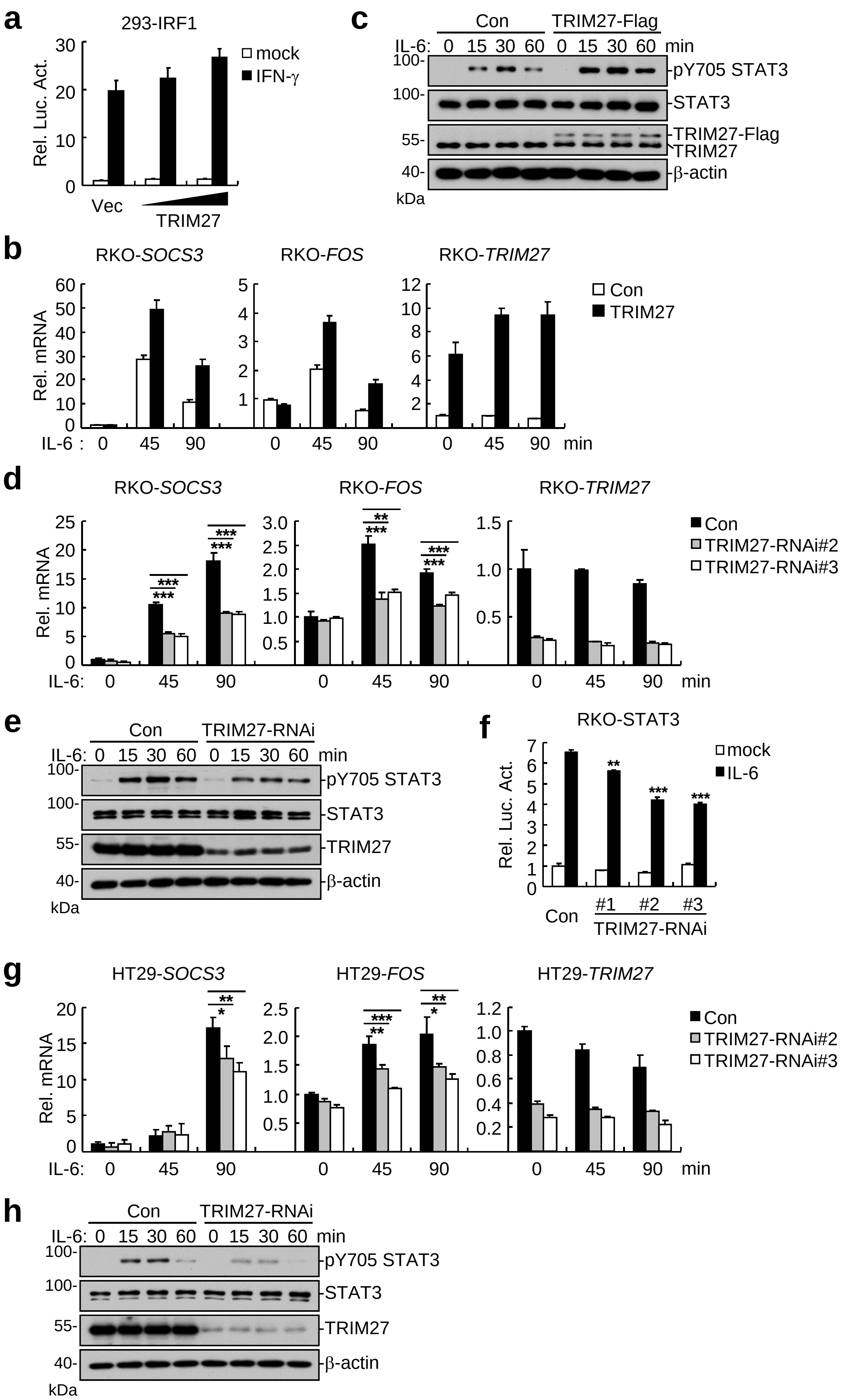

Supplementary Figure 1. TRIM27 potentiates IL-6-induced STAT3 activation in colonic epithelial cells, related to Figure 1. a Effects of TRIM27 on IFN-γ-induced IRF1 activation in HEK293 cells. **b-h** Effects of TRIM27 overexpression (**b&c**) and knockdown (**d-h**) on IL-6-induced transcription of *SOCS3* and *c-FOS* genes (**b**, **d** and **g**), STAT3 phosphorylation (**c**, **e** and **h**) and STAT3 activation (**f**) in RKO cells (**b-f**) and HT29 cells (**g&h**). Graphs show mean ± SD; n = 3. *P <0.05, **P < 0.01, ***P < 0.001, unpaired t test.

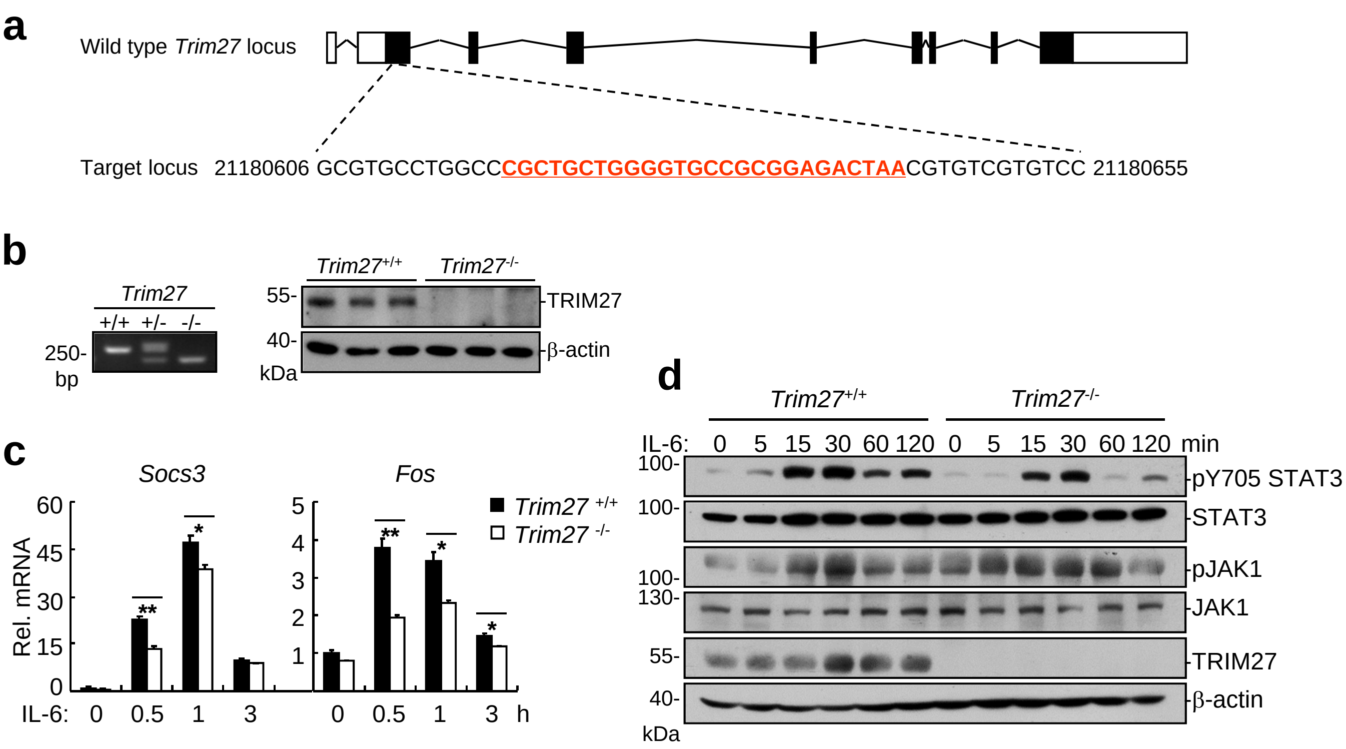

Supplementary Figure 2. TRIM27-deficiency inhibits IL-6–induced activation, related to Figure 1.
a Generation of *Trim27* $^{-/-}$ mice. *Trim27* $^{-/-}$ mice bears a 26-bp deletion in its exon-2. **b** Genotyping of *Trim27* knockout mice by PCR (left panel) and immunoblotting analysis of TRIM27 expression in the indicated genotypes of MEFs (right panel). **c** TRIM27-deficieny inhibits IL-6-induced transcription of *Socs3* and *c-Fos* genes in primary hepatocytes. Primary hepatocytes (2×10^5) isolated from *Trim27*$^{+/+}$ and *Trim27*$^{-/-}$ mice were starved overnight and stimulated with IL-6 (15 ng/mL) for the indicated times before qPCR experiments. **d** TRIM27-deficiency inhibits IL-6-induced STAT3 phosphorylation in primary hepatocytes. Primary hepatocytes (2×10^5) isolated from *Trim27*$^{+/+}$ and *Trim27* $^{-/-}$ mice were starved overnight and stimulated with IL-6 (15 ng/mL) for the indicated times before immunoblotting analysis was performed. Graphs show mean ± SD; n = 3. *P <0.05, **P < 0.01, ***P < 0.001, unpaired t test.

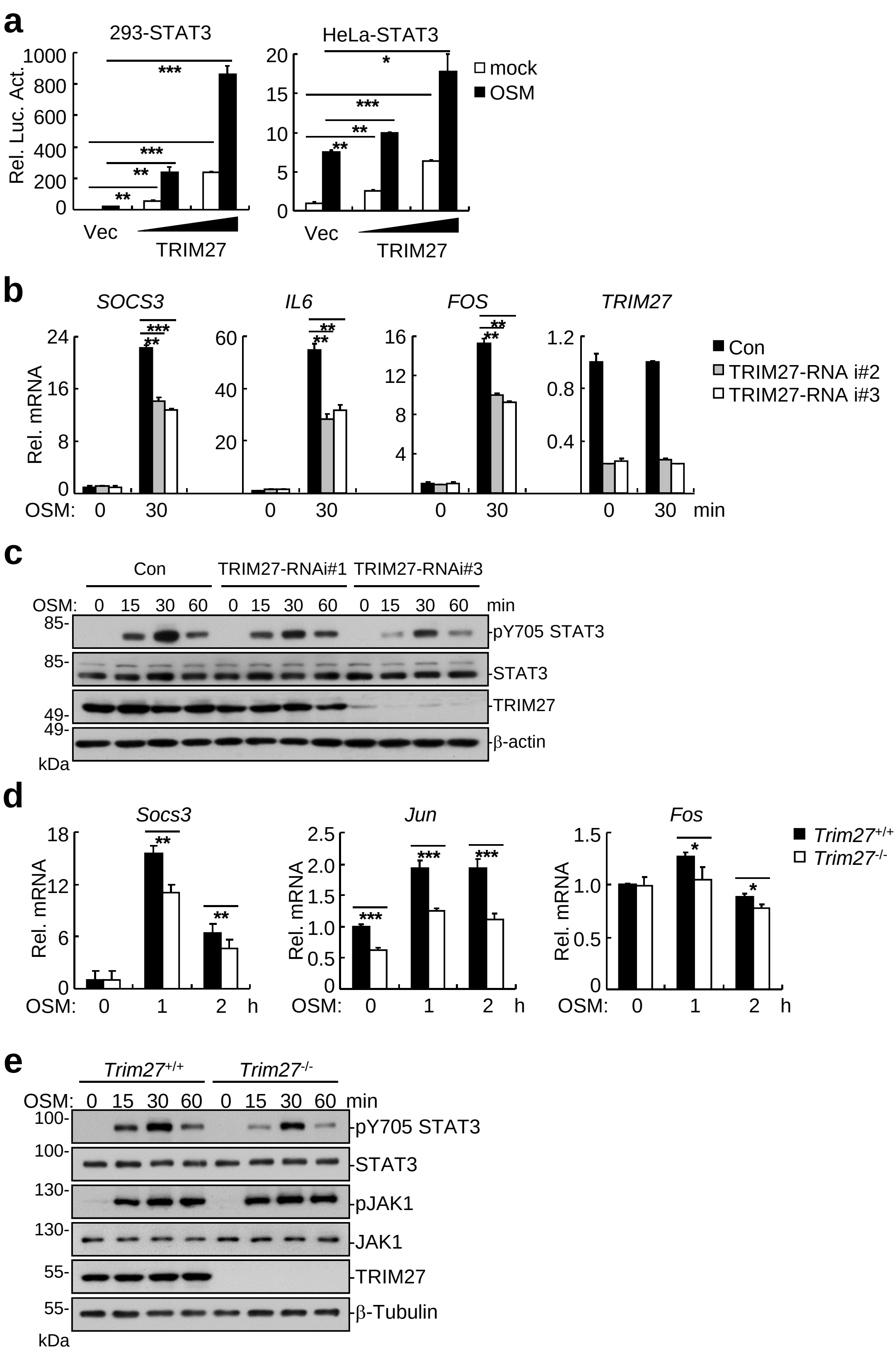

Supplementary Figure 3. TRIM27 activates STAT3 and potentiates OSM-induced STAT3 activation, related to Figure 1. a Effects of TRIM27 on OSM-induced STAT3 activation. **b&c** Effects of TRIM27 knockdown on OSM-induced transcription of downstream genes (**b**) and STAT3 phosphorylation (**c**). The control and TRIM27-RNAi cells (2×10^5) were starved overnight and stimulated with OSM (10 ng/mL) for the indicated times before qPCR experiments (**b**) and immunoblotting analysis (**c**) was performed. **d&e** Effects of TRIM27-deficiency on OSM-induced transcription of downstream (**d**) and STAT3 phosphorylation (**e**) in BMDM. *Trim27*$^{+/+}$ and *Trim27* $^{-/-}$ BMDMs (2×10^5) were starved overnight and stimulated with OSM (25 ng/mL) for the indicated times before qPCR experiments (**d**) immunoblotting analysis (**e**) was performed. Graphs show mean ± SD; n = 3. *P <0.05; **P < 0.01; ***P < 0.001, unpaired t test.

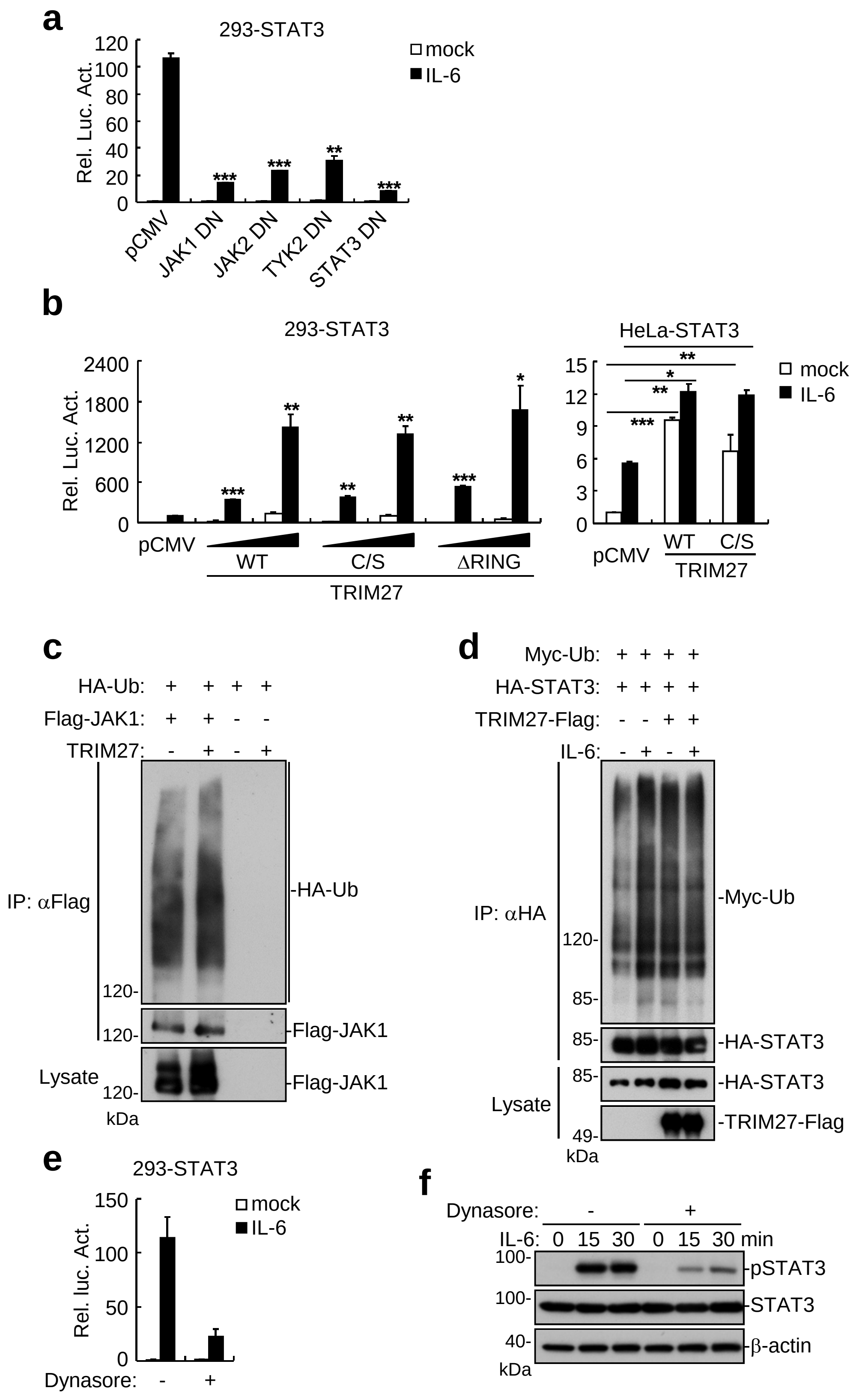

Supplementary Figure 4. TRIM27 activates STAT3 independent of its E3 ubiquitin ligase activity, related to Figure 2 and Figure 3. a Effects of various dominant negative mutants on IL-6-induced STAT3 activation. **b** Effects of wild-type and enzymatic inactive mutants of TRIM27 on IL-6-induced STAT3 activation. **c&d** Effects of TRIM27 on ubiquitination of JAK1 (**c**) and STAT3 (**d**). HEK293 cells were transfected with the indicated plasmids for twenty hours and treated with IL6 (50 ng/mL) or left untreated for 30 min before immunoprecipitation and immunoblotting analysis were performed. **e&f** Effects of dynasore treatment on IL-6-induced STAT3 activation (**e**) and STAT3 phosphorylation (**f**). The HEK293 cells were treated with dynasore (80 μM) for 1 h followed by IL-6 (20 ng/mL) treatment for 10 h before luciferase reporter assays (**e**) or for indicated time before immunoblotting analysis (**f**). Graphs show mean ± SD; n = 3. *P <0.05, **P < 0.01, ***P < 0.001, unpaired t test.

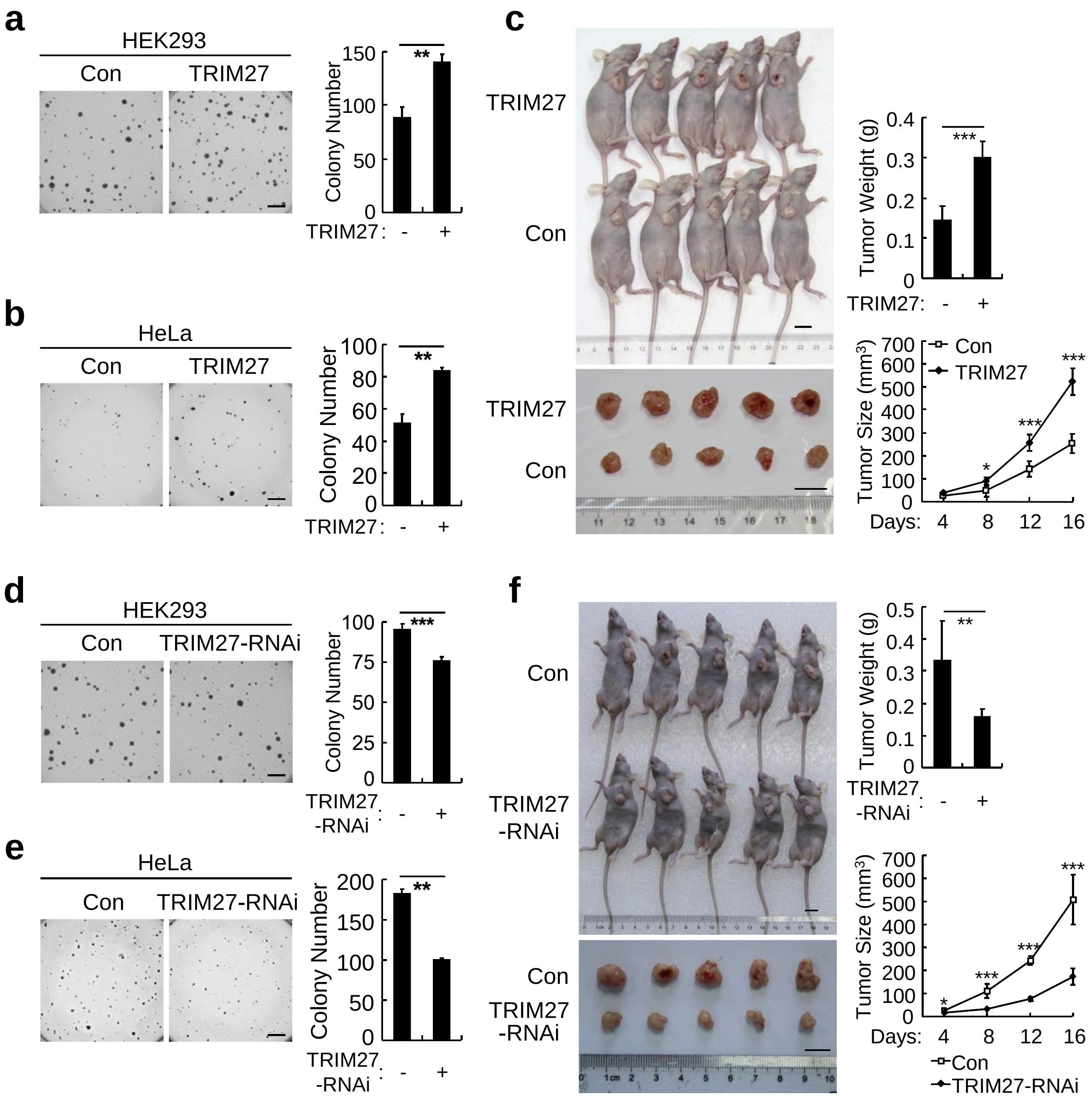

Supplementary Figure 5. TRIM27 promotes growth and tumorigenicity of tumor cells, related to Figure 5. **a**, **b**, **d** and **e** Effects of TRIM27 overexpression and knockdown on cell anchorage-independent growth. The control and TRIM27-overexpressing HEK293 cells or HeLa cells ($1x10^3$), the GFP-RNAi control and TRIM27-RNAi HEK293 cells ($1x10^3$) or HeLa cells ($2x10^3$),) were seeded in soft agar in 6-well plates. After 3 weeks, the colonies were photographed, and colony numbers were counted in each well. **c**&**f** Effects of TRIM27 overexpression and knockdown on tumor growth in nude mice. The control and TRIM27-overexpressing HeLa cells ($1x10^6$), GFP-RNAi control and TRIM27-RNAi HeLa cells ($2x10^6$) were injected into the flanks of nude mice. Mice were sacrificed and photographed at 16 days after injection and the tumor weights were measured. The tumor sizes were measured at an interval of 4 days after injection and calculated as $V=1/2xLxW^2$, where L and W represent the length and the width of the tumor respectively. Scale bars, 3 mm (**a**, **b**, **d** *and* **e**), 1 cm (**c**&**f**). Results are represented as mean ± SD, n=3 (**a**, **b**, **d** *and* **e**), n=5 (**c**&**f**). *P <0.05, **P < 0.01, ***P < 0.001, unpaired t test.

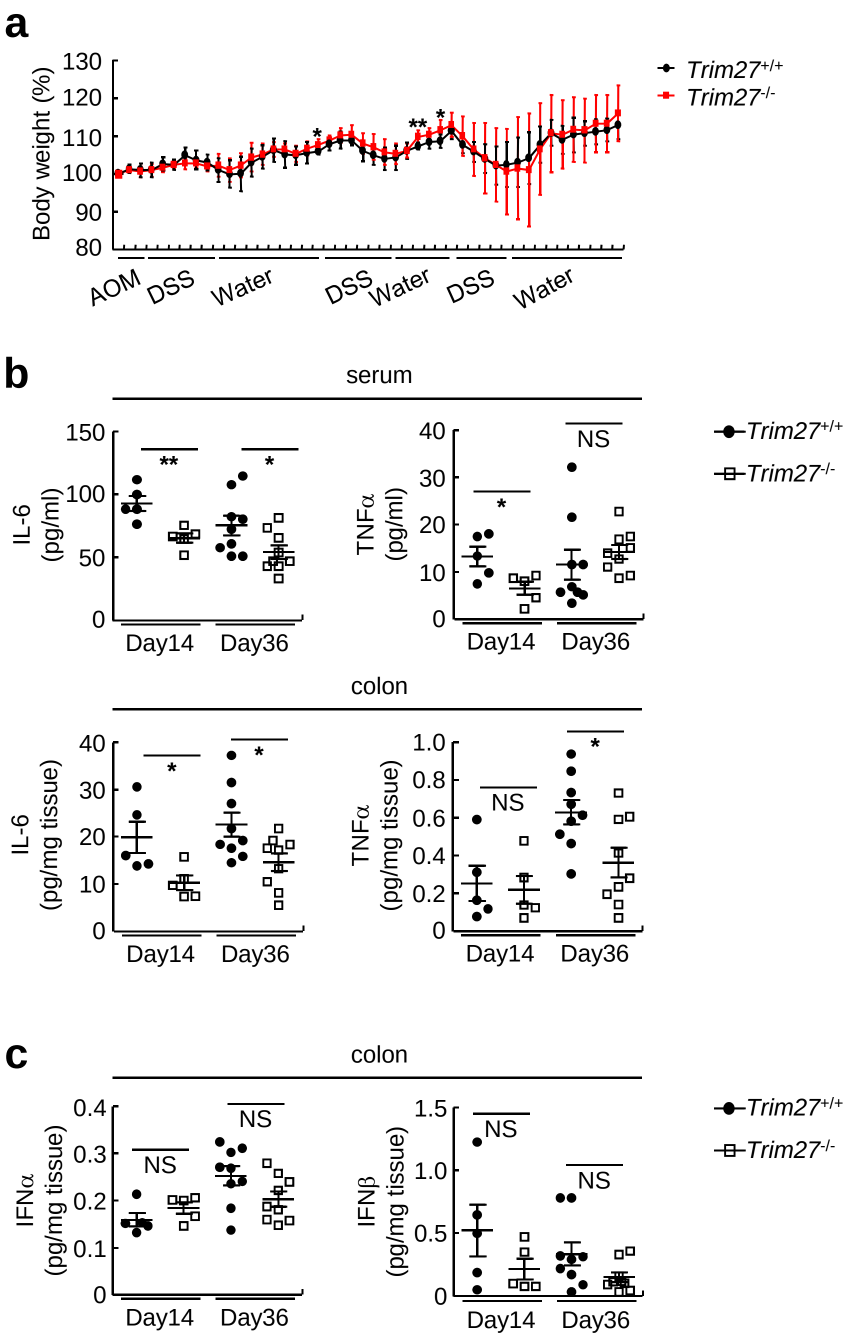

Supplementary Figure 6. Body weight changes and cytokines production of *Trim27*$^{+/+}$ and *Trim27* $^{-/-}$ mice during CAC development, related to Figure 8.

a *Trim27*$^{+/+}$ and *Trim27* $^{-/-}$ mice were treated as in the schematic diagram shown in **Figure 8a**. The body weights were daily measured during the procedures. **b** ELISA measurement of cytokine levels in sera and colon tissues of *Trim27*$^{+/+}$ and *Trim27* $^{-/-}$ mice treated with AOM/DSS for 14 days or 36 days. Results are shown as mean ± SD, n=5-9, *p < 0.05, **p < 0.01, unpaired t test. **c** ELISA measurement of type I interferons (IFN-αs and IFN-β) in colon tissues of *Trim27*$^{+/+}$ and *Trim27* $^{-/-}$ mice treated with AOM/DSS for 14 days and 36 days. Results are shown as mean ± SD, n=5-9.

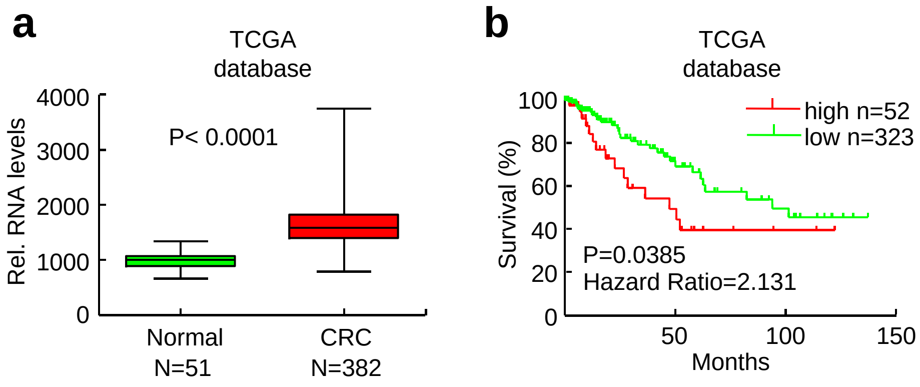

Supplementary Figure 7. TRIM27 level is up-regulated in human CRC samples.
a TRIM27 levels in human colorectal cancers. TRIM27 mRNA expression levels were evaluated in human colorectal cancers from the TCGA database. Significance was performed using Mann-Whitney *U* test. The horizontal lines in the box plots represent the median, the boxes represent the interquartile range and the whiskers represent the min and max values. **b** Correlation of TRIM27 levels with survival of human colorectal cancer patients. Kaplan-Meier curves for TRIM27 levels in association with survival of colorectal cancer patients.

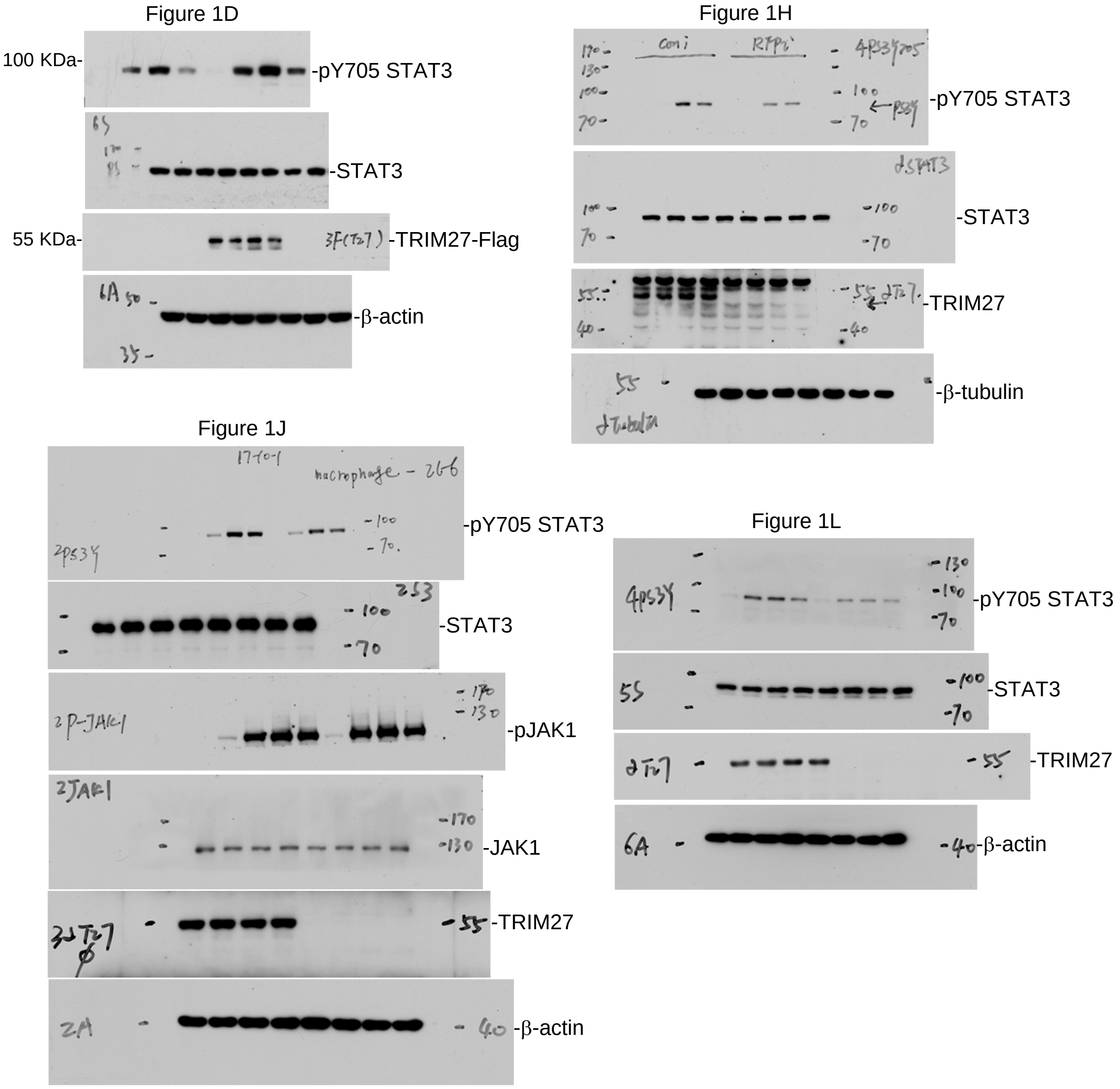

Supplementary Figure 8. Un-cropped blots for Figure 1 d, h, j and l.

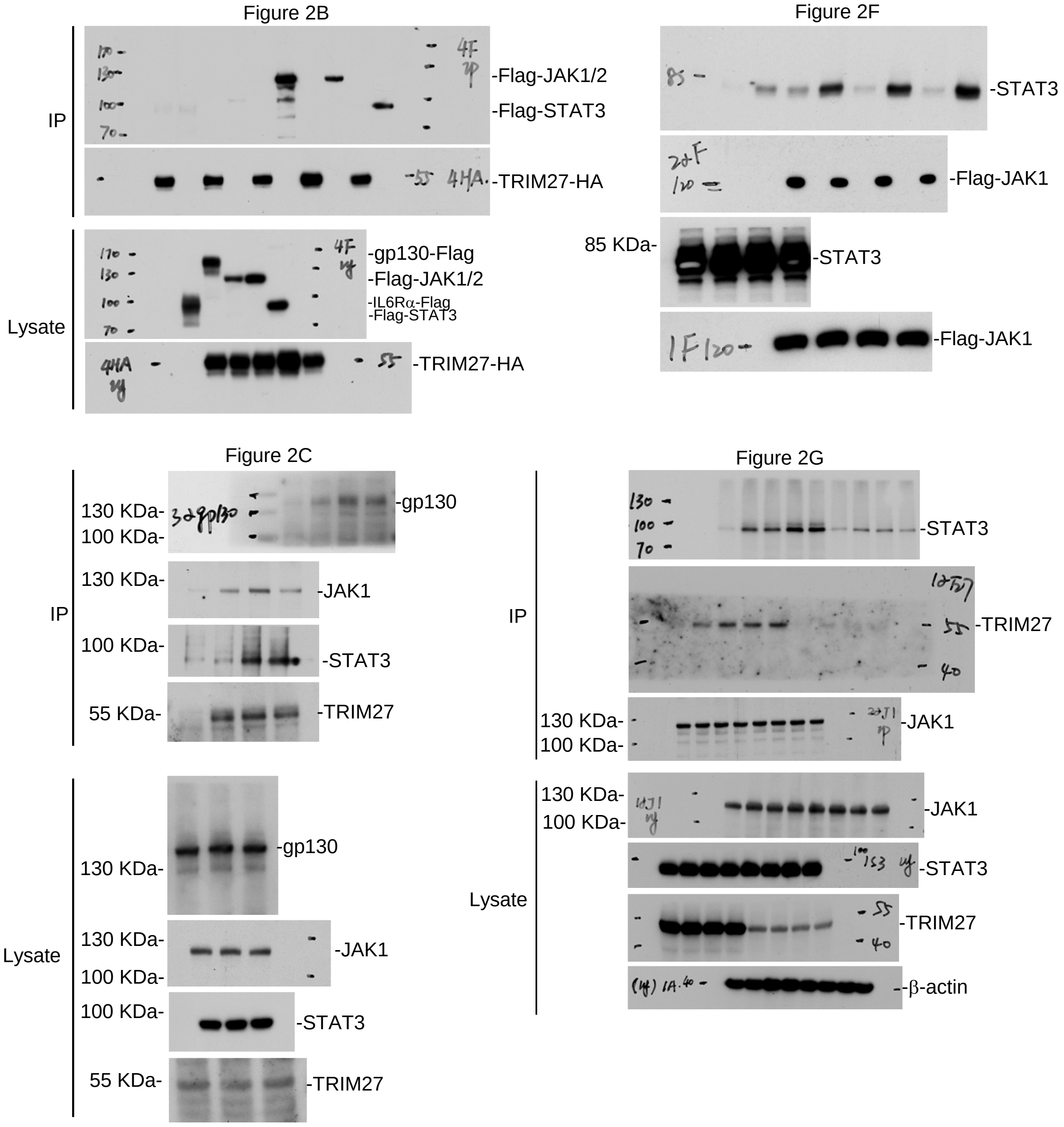

Supplementary Figure 9. Un-cropped blots for Figure 2 b, c, f and g.

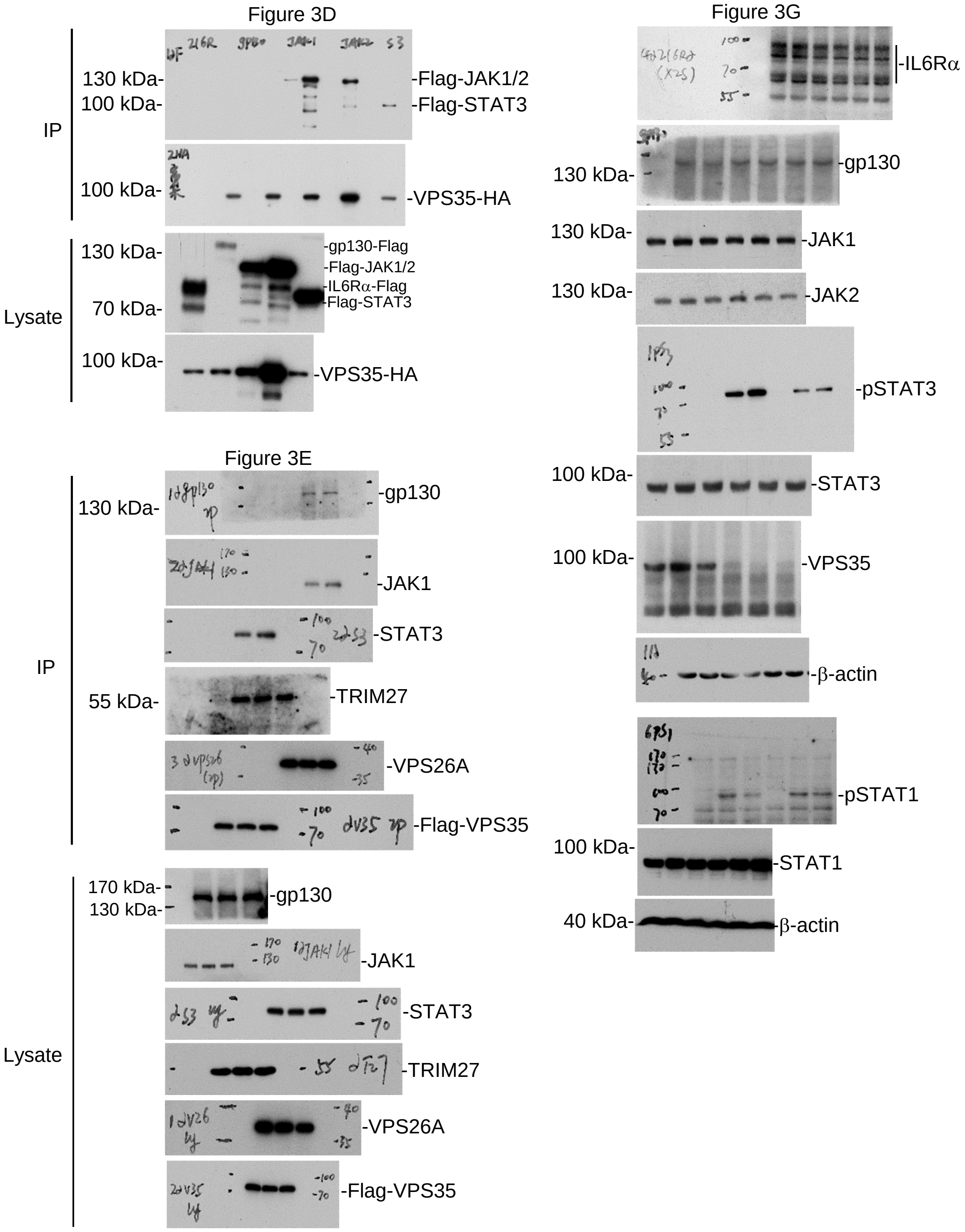

Supplementary Figure 10. Un-cropped blots for Figure 3d, e and g.

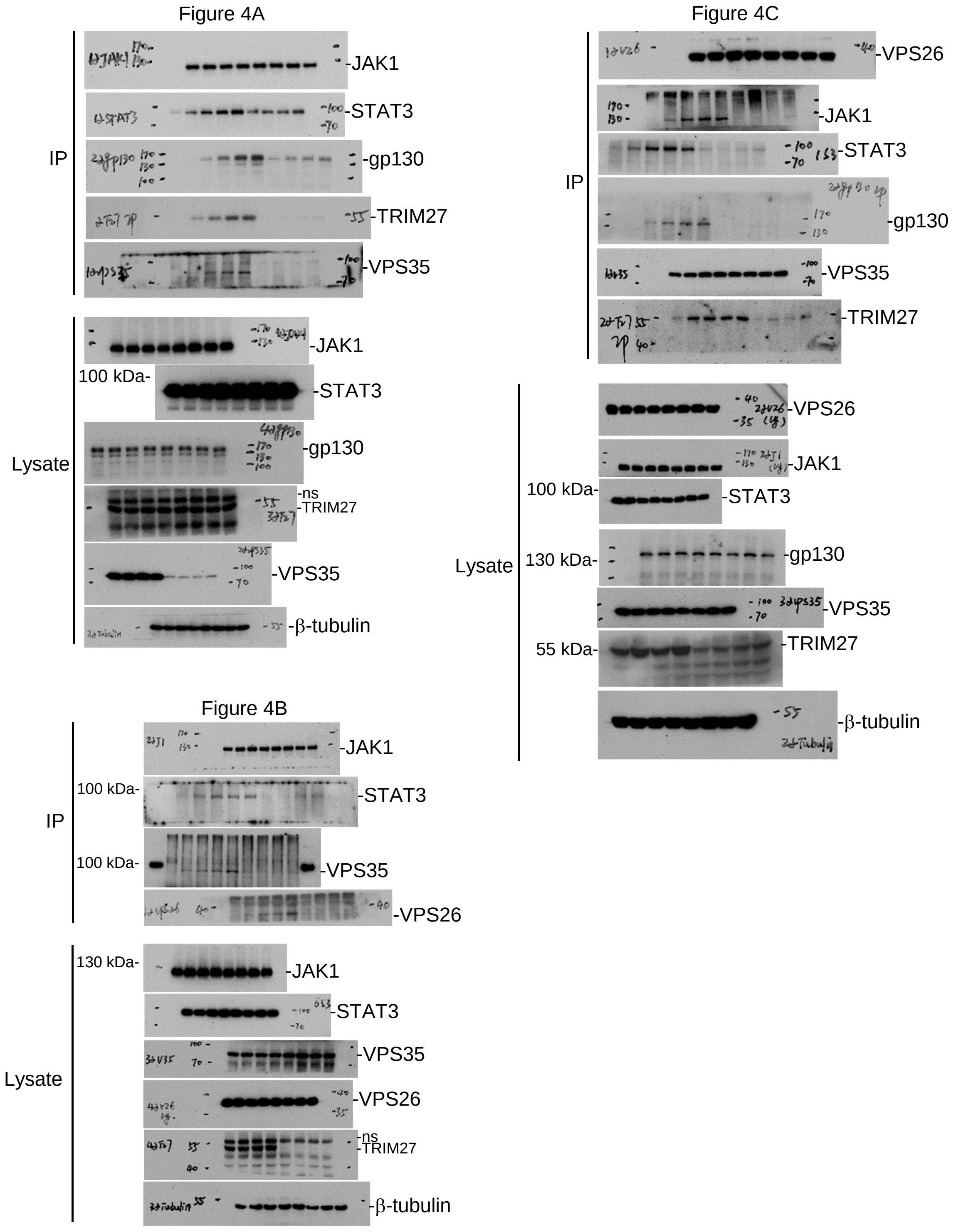

Supplementary Figure 11. Un-cropped blots for Figure 4a, b and c.

Figure 6f

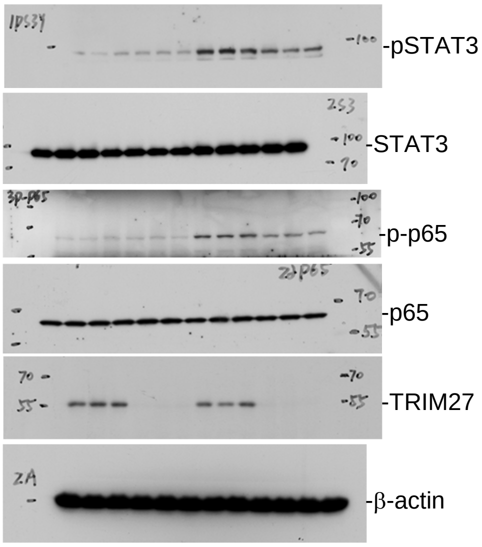

Figure 7f

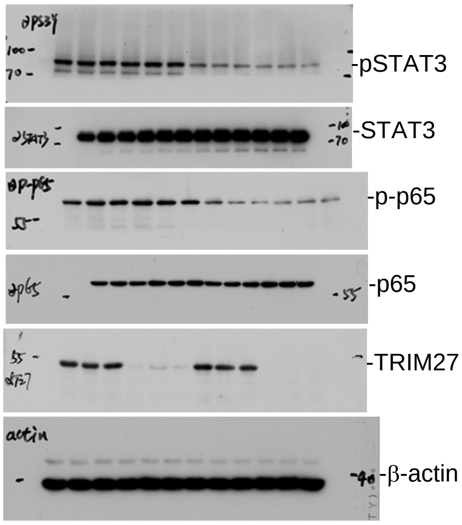

Figure 8d

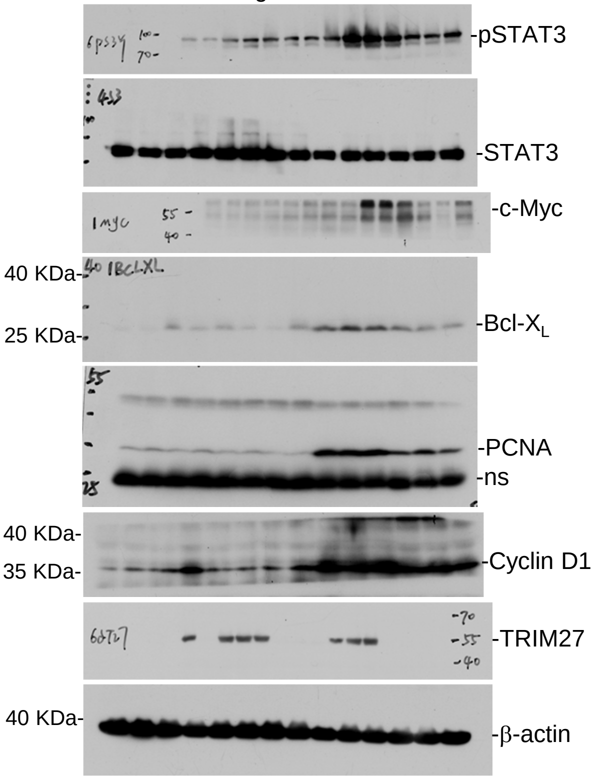

Supplementary Figure 12. Un-cropped blots for Figure 6f, 7f and 8d.

Supplementary Figure 1c

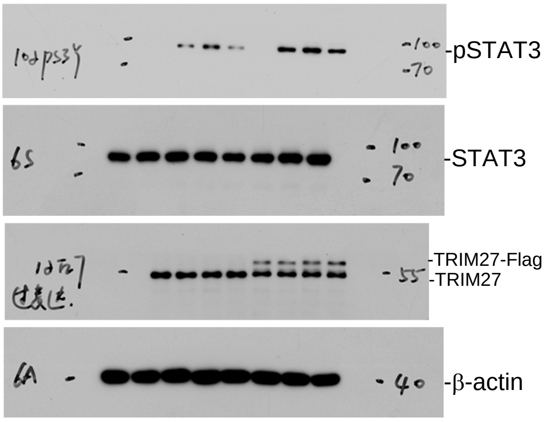

Supplementary Figure 1e

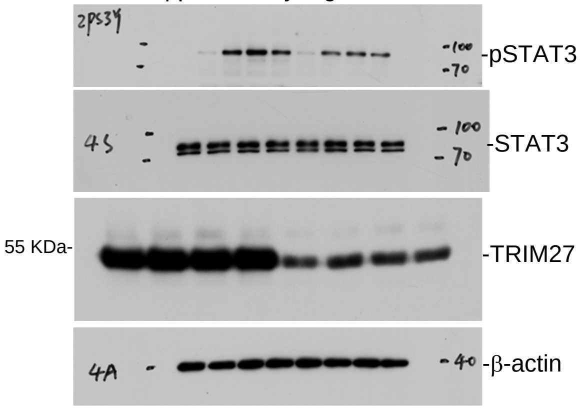

Supplementary Figure 1h

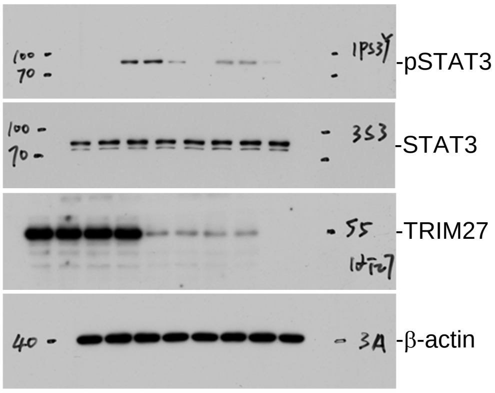

Supplementary Figure 2b

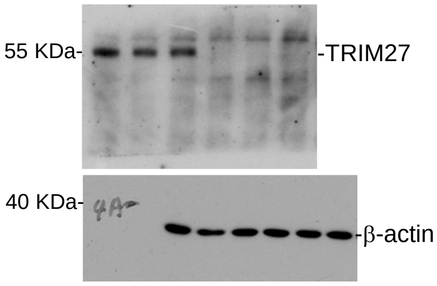

Supplementary Figure 2d

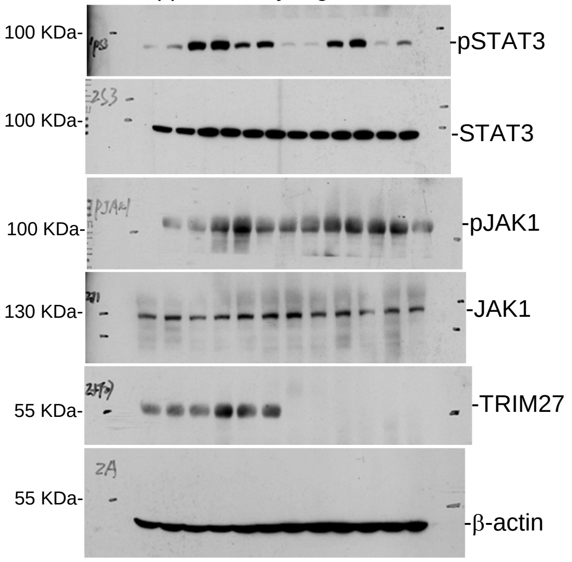

Supplementary Figure 13. Un-cropped blots Supplementary Figure 1c, 1e, 1h, 2b and 2d.

Supplementary Figure 3c

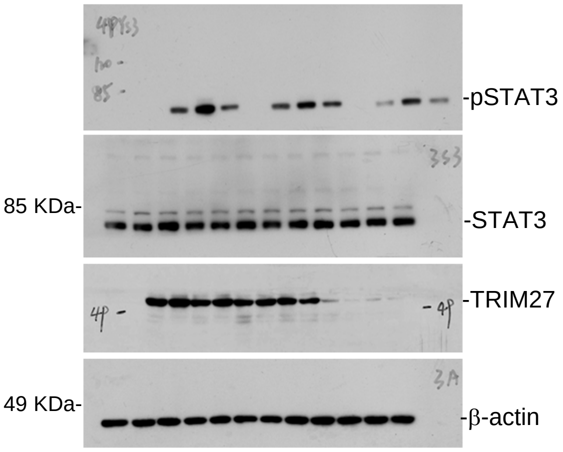

Supplementary Figure 3e

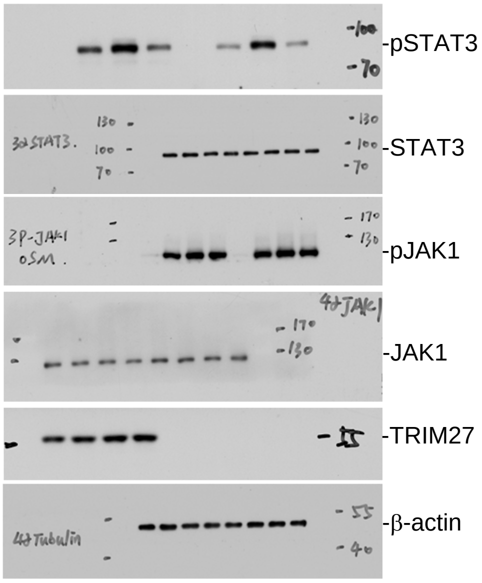

Supplementary Figure 4f

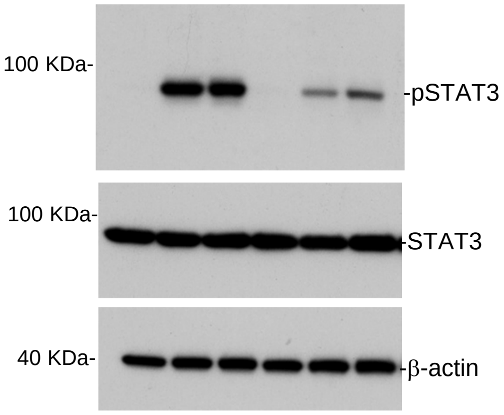

Supplementary Figure 4c

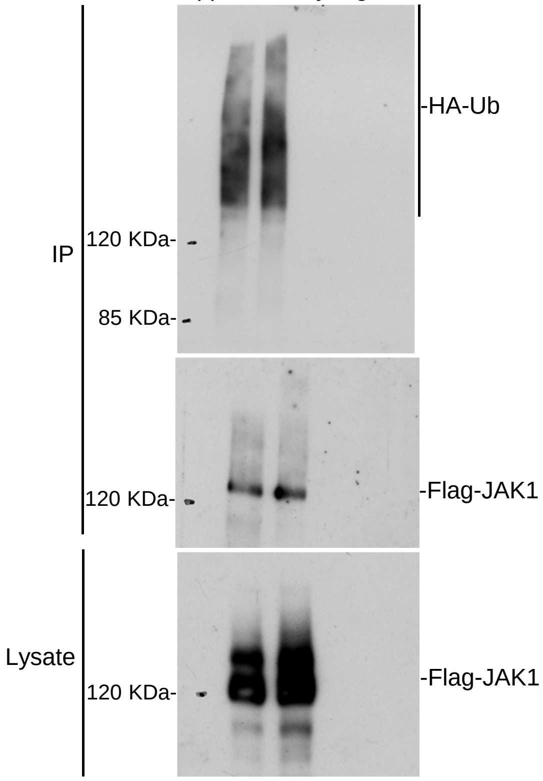

Supplementary Figure 4d

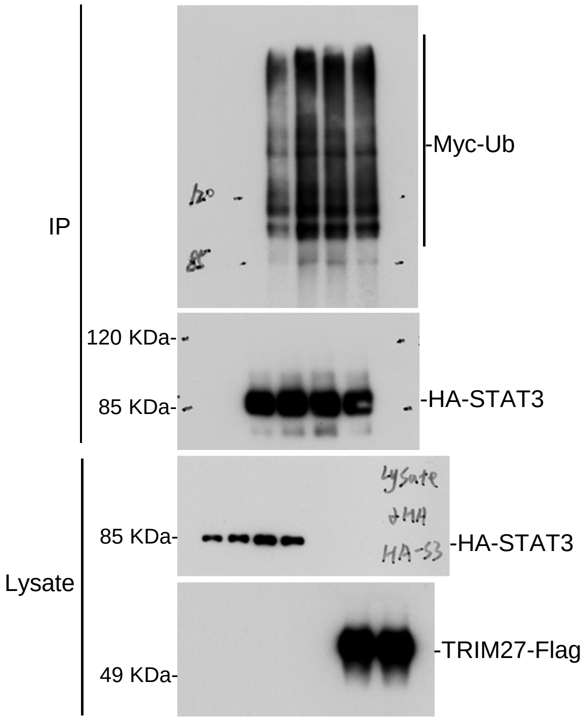

Supplementary Figure 14. Un-cropped blots for Supplementary Figure 3e, 3e, 4e, 4d and 4f.

Supplementary Table 1. Primers used in qPCT experiments.

Gene	Forward sequence	Reverse sequence
GAPDH	5’-CGGAGTCAACGGATTTGGTCG-3’	5’-AGCCTTCTCCATGGTGGTGAAG-3’
IL-6	5’-TTCTCCACAAGCGCCTTCGGTC-3’	5’-TCTGTGTGGGGCGGCTACATCT-3’
SOCS3	5’-CATCTCTGTCGGAAGACCGTCA-3’	5’-GCATCGTACTGGTCCAGGAACT-3’
c-FOS	5’-GCCTCTCTTACTACCACTCACC-3’	5’-AGATGGCAGTGACCGTGGGAAT-3’
TRIM27	5’-TGCTGGTGAGGTCTCCTTCT-3’	5’-CTCAGACTGAAGTAGGGCCG-3’
VPS35	5’-GTCAAGTCATTTCCTCAGTCCAG-3’	5’-CCCCTCAAGGGATGTTGCAC-3’
VPS26	5’-TCAGGAAAGGTAAACCTAGCCTT-3’	5’-ATTGGCACCGATGTAAGATTCAT-3’
VPS29	5’-CTCAAGACTCTGGCTGGTGATG-3’	5’-CTGTCCAACAGTCACAACTTTCTG-3’
Gapdh	5’-ACGGCCGCATCTTCTTGTGCA-3’	5’-ACGGCCAAATCCGTTCACACC-3’
Il-6	5’-TCTGCAAGAGACTTCCATCCAGTTGC-3’	5’-AGCCTCCGACTTGTGAAGTGGT-3’
Socs3	5’-GGACCAAGAACCTACGCATCCA-3’	5’-CACCAGCTTGAGTACACAGTCG-3’
c-Fos	5’-GGGAATGGTGAAGACCGTGTCA-3’	5’-GCAGCCATCTTATTCCGTTCCC-3’
Trim27	5’-AGCCTCTGAAGCTGTACTGCGA-3’	5’-CTTAGGTGGTCCAGTCGGTTCT-3’
Tnfa	5’-GGTGATCGGTCCCCAAAGGGATGA-3’	5’-TGGTTTGCTACGACGTGGGCT-3’
Il-17a	5’-CATGAGTCCAGGGAGAGCTT-3’	5’-ATCTATCAGGGTCTTCATTGCGG-3’
